# Case report: A novel *de novo* germline loss-of-function mutation in the STAT1 transactivation domain in two Chinese siblings, with the elder sibling presenting with multifocal *Bacillus* Calmette–Guerin osteomyelitis

**DOI:** 10.3389/fimmu.2024.1504816

**Published:** 2025-01-07

**Authors:** Qin Ying Lim, Daniel Leung, Crystal K. Lam, Xingtian Yang, Kai N. Cheong, Andrew K. H. Yik, Jing Yang, Koon-Wing Chan, Pamela P. W. Lee, Miyuki Tsumura, Elaine Y. L. Au, Jaime S. Rosa Duque, Satoshi Okada, Yu Lung Lau

**Affiliations:** ^1^ Department of Paediatrics and Adolescent Medicine, Queen Mary Hospital, Hong Kong, Hong Kong SAR, China; ^2^ Department of Paediatrics and Adolescent Medicine, The University of Hong Kong, Hong Kong, Hong Kong SAR, China; ^3^ Division of Clinical Immunology, Department of Pathology, Queen Mary Hospital, Hong Kong, Hong Kong SAR, China; ^4^ Department of Paediatrics and Adolescent Medicine, Hong Kong Children’s Hospital, Hong Kong, Hong Kong SAR, China; ^5^ Department of Paediatrics, The University of Hong Kong – Shenzhen Hospital, Shenzhen, China; ^6^ Department of Paediatrics, Hiroshima University Graduate School of Biomedical and Health Sciences, Hiroshima, Japan

**Keywords:** STAT1 loss-of-function, BCG osteomyelitis, Mendelian susceptibility to mycobacterial diseases, germline mosaicism, case report

## Abstract

Signal transducer and activator of transcription 1 (STAT1) gene mutations have broad clinical phenotypes, classified by the inheritance pattern and functional state. Individuals with autosomal dominant STAT1 deficiency are more susceptible to intracellular bacteria, the hallmark of which is Mendelian susceptibility to mycobacterial diseases (MSMDs) that are associated with increased risks of invasive disease by weakly virulent mycobacteria. We report a novel *de novo* heterozygous missense mutation in exon 23 of the STAT1 gene (NM_007315.4):c.2129C>T(p.Ser710Phe) (S710F), located in the transactivation domain (TAD) for two Chinese siblings, whereby the index patient presented with multifocal osteomyelitis after *Bacillus* Calmette–Guerin (BCG) vaccine, while the younger sibling was spared the infection, as BCG vaccination was withheld at birth. STAT1 loss-of-function was confirmed by the gamma-activated sequence reporter assay, representing the first loss-of-function mutation in the TAD of the STAT1 gene. Both parents did not have the same mutation, and this finding is suggestive of gonadal mosaicism.

## Introduction

Signal transducer and activator of transcription 1 (STAT1) is an important molecule involved in interferon (IFN)-mediated immunity as part of the well-known Janus activation kinases (JAK)-STAT pathway. IFN-γ stimulation leads to phosphorylation of the STAT1 tyrosine 701 residue (Y701) by Janus kinase 1 and 2 in the cytoplasm, forming the IFN-γ activated factor (GAF) homodimer. The homodimer is translocated into the nucleus, where it binds to the gamma-activated sequence (GAS) to induce transcription of target genes involved in defense against intracellular pathogens. In contrast, stimulation by IFN-α and IFN-β induces phosphorylation of STAT1 and STAT2 that bind to interferon regulating factor 9 (IRF9) to form the IFN-stimulated gene factor 3 (ISGF3) heterotrimer. The heterotrimer translocates into the nucleus and binds to the IFN-stimulated response element (ISRE) to induce target gene transcription involved in defense against viruses. This extensive signaling and activation pathway that mediate regulation of IFN-responsive target gene transcription play a crucial role in human immune defense (1).

Over 150 disease-causing STAT1 gene mutations with broad clinical phenotypes have been described, classified by the inheritance pattern and functional state (2). STAT1 loss-of-function diseases include autosomal dominant (AD) STAT1 deficiency and autosomal recessive (AR) complete and partial STAT1 deficiency. The hallmark of AD STAT1 deficiency is Mendelian susceptibility to mycobacterial diseases (MSMD), and individuals with this disease are susceptible to invasive infections caused by weakly virulent mycobacteria (3). Those with AR STAT1 deficiency have increased susceptibility to intracellular organisms and viruses in addition to increased susceptibility to mycobacteria, of which AR complete STAT1 deficiency often has more severe clinical phenotypes compared to AR partial STAT1 deficiency. On the other hand, those with AD STAT1 gain-of-function (GOF) mutations present with chronic mucocutaneous candidiasis (CMC) (4).

We present a pair of Chinese siblings with novel *de novo* heterozygous missense mutation in exon 23 of the STAT1 gene, which was identified after the index patient presented with multifocal osteomyelitis following *Bacillus* Calmette–Guerin (BCG) vaccination. Subsequently, his younger sister was found to have the same mutation on family screening while the parents were unaffected, suggesting gonadal mosaicism. This mode of inheritance is seldom recognized or reported in patients with inborn errors of immunity (IEI), which can lead to missed diagnoses. The mutation is the first reported loss-of-function STAT1 located in the transactivation domain (TAD).

## Case description

### Patient 1

**Figure 1:**
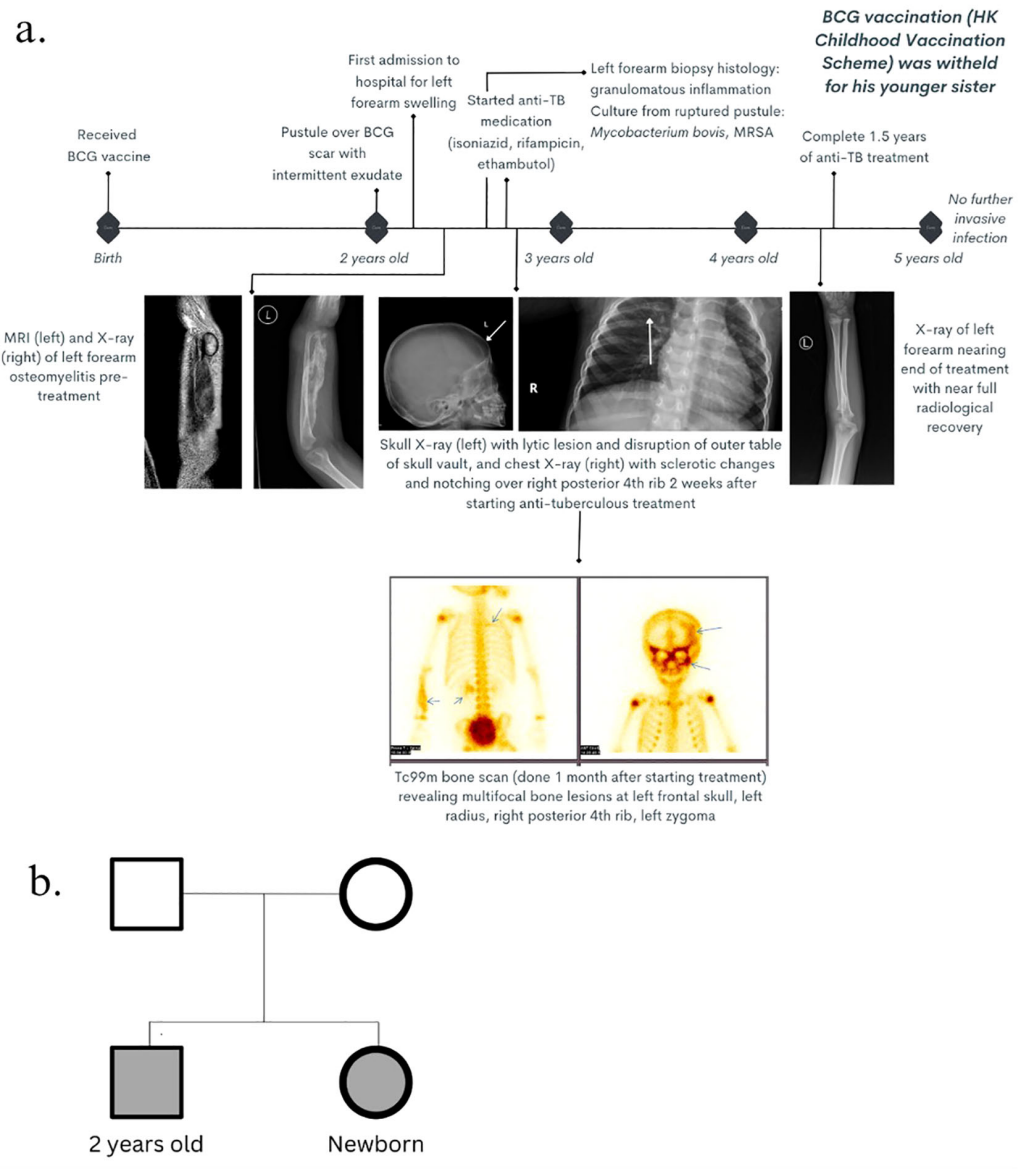
Clinical history. **(A)** Timeline of clinical course, radiological features, and management from birth until 5 years old. **(B)** Genogram of the two patients with unaffected parents. 
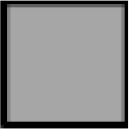
: male (affected); 
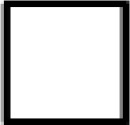
: male (unaffected); 
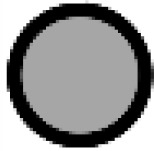
: female (affected); 
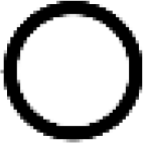
: female (unaffected).

Patient 1 was a 2-year-old Chinese boy with non-consanguineous parents and no personal or family history of recurrent infections or early neonatal death, who presented with left wrist and forearm swelling in the past 2 months. Additionally, there was history of pustule formation over his neonatal intradermally administered live-attenuated *Mycobacterium bovis* Bacille Calmette–Guerin (BCG) vaccine scar site on his left upper arm and, thereafter, intermittent discharge from the same site for approximately 1 year. The patient otherwise remained well and had satisfactory growth parameters, with no fever, chronic respiratory symptoms, unusual environmental exposures, or challenges in accessing healthcare since birth (**Figure 1A**). His parents had no history of mycobacterial infections.

### Patient 2

Patient 2 was patient 1’s newborn younger sister (**Figure 1B**).

## Diagnostic assessment

### Patient 1

Ultrasonography showed a non-vascular left distal radial lesion, with osseous and soft tissue component changes, and axillary lymphadenopathy, consistent with osteomyelitis of low-grade infection. X-ray of the left forearm showed features of acute osteomyelitis. Magnetic resonance imaging (MRI) of the left forearm was performed 4 months later, which was delayed due to his inadequate fasting period required for sedation and the waiting queue for public radiological services in this region, and the findings confirmed acute osteomyelitis and sinus formation. Further assessment with MRI of the brain and bone scan revealed disseminated osteomyelitis involving the skull, left zygoma and right posterior fourth rib (**Figure 1B**). Biopsy of the left radius was taken, and the histological examination revealed focal granuloma formation, dense lymphoplasmacytic infiltrates, scattered eosinophils, and multinucleated giant histiocytes engulfing the calcified and transparent foreign material, consistent with a granulomatous lesion, while the microbiological culture yielded *Mycobacterium bovis*. Culture of the purulent discharge from the ulcerated BCG injection site was positive for attenuated *Mycobacterium bovis* and *methicillin-resistant Staphylococcus aureus* (MRSA).

Immunological workup was not suggestive of chronic granulomatous disease (CGD); the neutrophil function by nitroblue tetrazolium test (NBT) was normal, and although the initial dihydrorhodamine reduction test (DHR) performed during the initial infection showed suppressed oxidative burst activity with low mean fluorescent intensity (MFI), oxidative burst activity normalized on repeat testing. The lymphocyte subset was not suggestive of severe combined immunodeficiency (SCID). The clinical or biochemical condition was not consistent with hemophagocytic lymphohistiocytosis.

Subsequent to these initial diagnostic testing methods, genetic workup by whole exome sequencing (WES) to survey for disease-causing coding genes, followed by confirmation using Sanger sequencing (SS), found a novel *de novo* heterozygous missense mutation in exon 23 of the STAT1 gene (NM_007315.4): c.2129C>T (p.Ser710Phe) (**Supplementary Material**), which has not been previously reported. The mutation was located in the transactivation domain of the STAT1 molecule.

### Patient 2

Patient 2 was identified upon further screening of immediate family members by targeted gene SS, which revealed the same STAT1 mutation (**Figure 1B**).

### Genetic investigation and functional testing

Although the clinical phenotype of patient 1 was compatible with AD STAT1 deficiency, the same STAT1 mutation by targeted gene SS was not found in either parent, suggestive of gonadal mosaicism. This variant found in the two siblings has a high pathogenicity score, with a combined annotation-dependent depletion (CADD) score of 6.988 and a deleterious annotation of genetic variants using neural networks (DANN) score of 0.999. The mutation is located at a highly conserved position, with a genomic evolutionary rate profiling (GERP) score of 5.95. According to the ACMG guidelines, the variant was classified as likely pathogenic.

**Figure 2:**
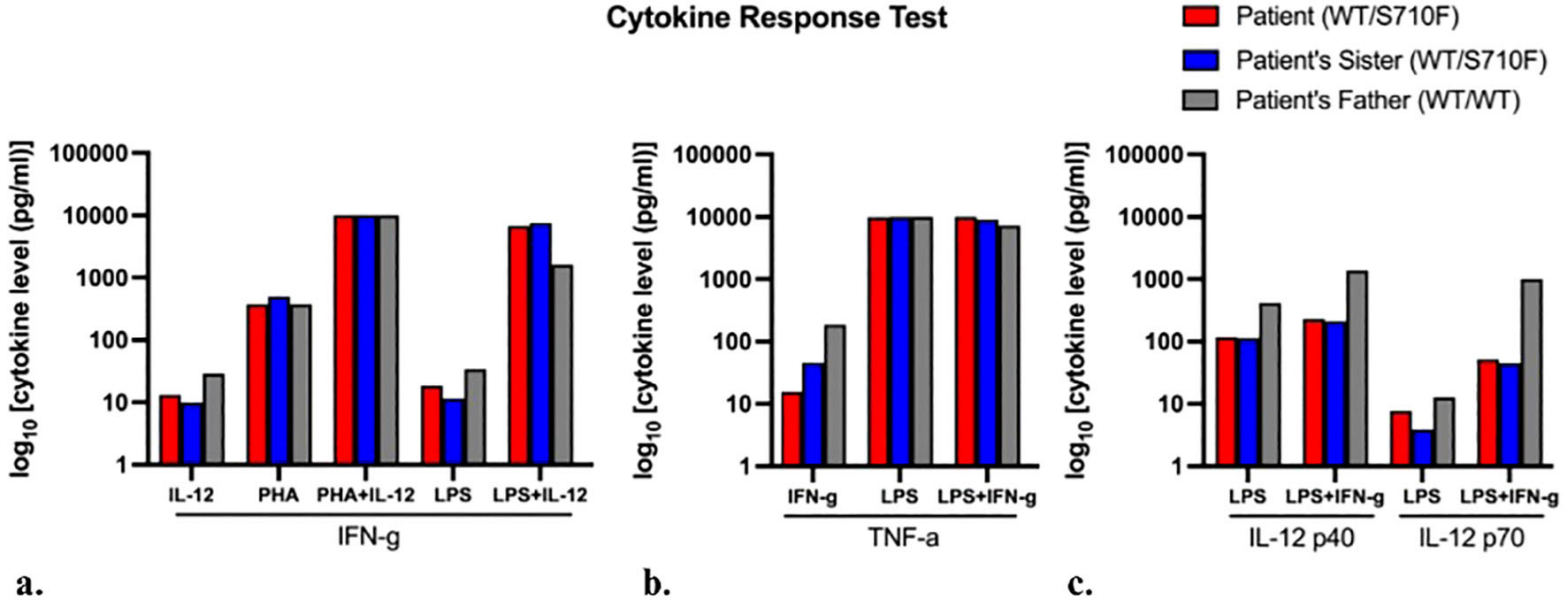
Cytokine response test. Cytokine response levels produced by blood mononuclear cells when stimulated by agents listed on the *x*-axis, in the production of **(A)** interferon-γ (IFN-γ), **(B)** tumor necrosis factor-α (TNF-α), and **(C)** interleukin 12 (IL-12), respectively. Levels in patient’s father (control sample) shown in gray. Each bar represents an individual’s single assay result; hence, statistical comparison was non-applicable.

In the cytokine response assay (**Supplementary Material**), there was normal response in the production of IFN-γ to IL-12 with or without phytohemagglutinin (PHA) or lipopolysaccharide (LPS) for both siblings (**Figure 2A**). However, the two siblings had reduced production of tumor necrosis factor alpha (TNF-α) with IFN-γ stimulation, which normalized with the addition of LPS (**Figure 2B**). These findings were supportive of IFN-γ response defect, such as IFN-γ receptor deficiency and STAT1 deficiency due to markedly reduced interleukin-12 (IL-12) p40 and p70 production in response to IFN-γ stimulation of peripheral blood mononuclear cells (PBMCs), which was more obvious in response to combined lipopolysaccharide (LPS) and IFN-γ (**Figure 2C**).

**Figure 3:**
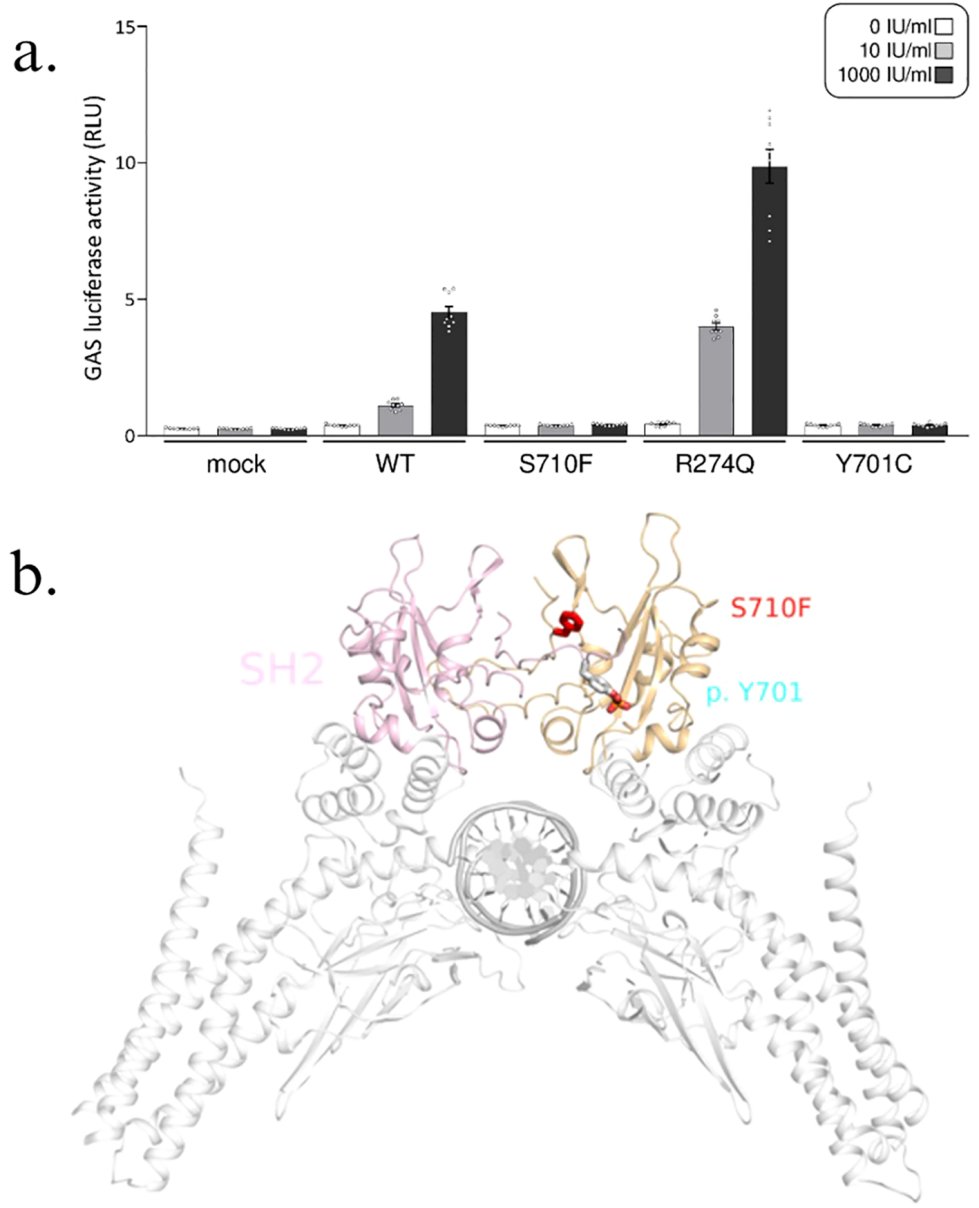
STAT1 S710F mutation. **(A)** Gamma-activated sequence (GAS) reporter assay with human cells completely lacking STAT1 (U3C cells) that transiently express wild-type (WT) or mutant STAT1 were subjected to a GAS reporter assay. Both the S710F (mutation in our patients) and Y701C (known loss-of-function mutation) STAT1 mutants exhibited reduced GAS activation, whereas the R274Q (known gain-of-function mutation) mutant resulted in increased GAS activation upon IFN-γ stimulation. This GAS reporter assay confirmed that the two siblings had a STAT1 loss-of-function mutation. The bar graphs display the means with standard errors. **(B)** STAT1 protein with the mutation site S710F (red); activation of STAT1 by phosphorylation at Y701 (blue).

The gamma-activated sequence (GAS) reporter assay was performed (**Supplementary Material**). The S710F mutant in our patients, along with Y701C, a known loss-of-function mutation identified in patients with MSMD (5), exhibited decreased activation of GAS compared to the wild type (WT) upon stimulation by IFN-γ (**Figure 3A**). In contrast, the R274Q mutant, a known gain-of-function mutation found in patients with chronic mucocutaneous candidiasis, showed excess GAS activation in response to IFN-γ.

## Therapeutic intervention

Patient 1 was treated for disseminated BCG osteomyelitis with a prolonged course of anti-mycobacterial regimen using isoniazid, rifampicin, and ethambutol for 1 year and 8 months.

BCG vaccination according to the Hong Kong Childhood Vaccination Scheme was withheld for patient 2 after her birth. She was advised to avoid high-risk mycobacterial environmental and sick-contact exposures, while pharmacological mycobacterial prophylaxis was deemed unnecessary.

## Follow-up and outcomes

Genetic counseling was provided to the family and long-term follow-up assessments to the two siblings.

**Figure 4:**
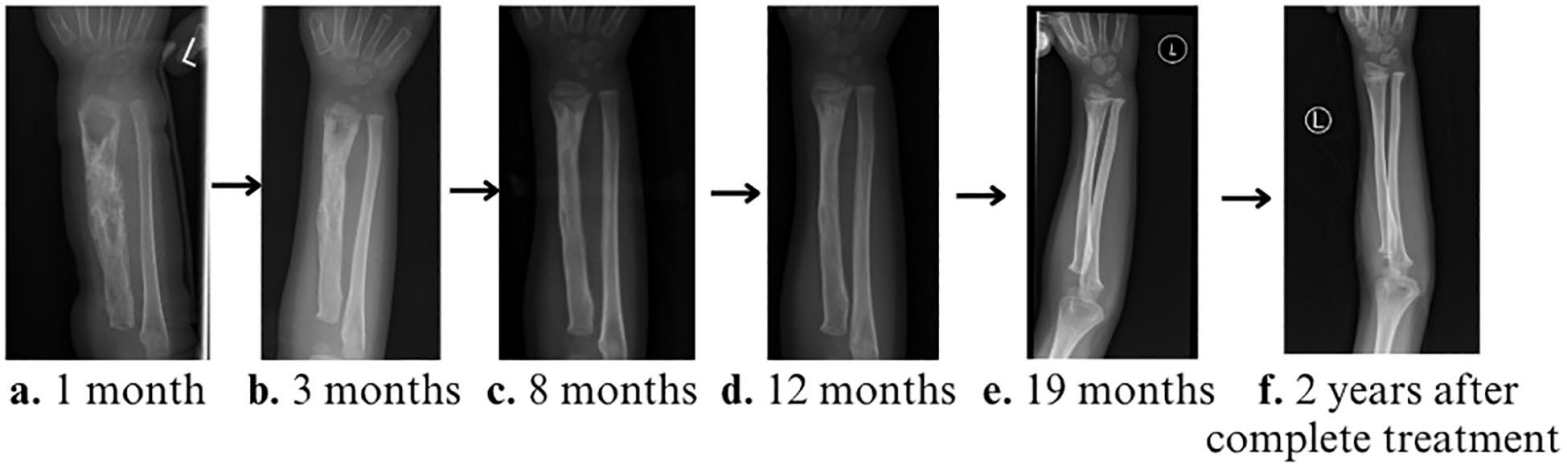
Serial X-rays of left forearm during treatment. **(A)** 1 month, **(B)** 3 months, **(C)** 8 months, **(D)** 12 months, and **(E)** 19 months (immediately prior to discontinuation of the anti-tuberculosis medications) showing progressive radiological disease improvement, with near full recovery and remodeling towards the end of the treatment. **(F)** X-ray of the left forearm 2 years after treatment completion, with full bone remodeling.

There was clinical and radiological recovery of osteomyelitis for patient 1 (**Figure 4**). He remains well with no further invasive infection. Although this young child was unable to attend nursery and kindergarten during his anti-tuberculous therapy, our directly observed therapy (DOT) outpatient service aimed at improving daily life, psychosocial wellbeing, and drug adherence facilitated early hospital discharge and adequate treatment assurance, for which the parents expressed gratitude in easing the patient’s diagnostic and treatment journey.

Although her older sibling had disseminated osteomyelitis, patient 2 has remained asymptomatic from birth to age 6 years at the time of her last clinical follow-up.

## Discussion

We identified unique features in these two siblings that had a novel *de novo* heterozygous loss-of-function STAT1 mutation associated with disseminated BCG osteomyelitis. This genetic mutation likely resulted from gonadal mosaicism, and to the best of our knowledge, the siblings also represent the first reported loss-of-function mutation located in the transactivation domain (TAD, residues 710–750) of the STAT1 gene. The two above features in these siblings have not been reported in STAT1 LOF mutations previously.

The STAT1 protein is comprised of the N-terminal domain, coiled-coil domain (CCD), DNA binding domain, linker domain, SH2 domain, and tail segment domain, in addition to the TAD. The CCD, involved in protein–protein interactions and the SH2 domain, which is required for STAT1 recruitment to interferon receptors (6, 7), had been more frequently implicated in STAT1 LOF mutations (Asano et al., 2023, Ye et al., 2022). These mutations can lead to impairment of STAT1 dimerization and dephosphorylation of pSTAT1 (8–11). To date, four other mutations located in the TAD have been reported, but all had been gain-of-function mutations (12). For our mutation, changes in local conformation of the STAT1 protein due to the proximity of the mutation site to the phosphorylation site (**Figure 3B**) may explain the effect on STAT1 phosphorylation. This can result in impaired downstream signaling of the GAF homodimer and its critical role in host defense against *Mycobacteria.* In addition, serine 727 phosphorylation of STAT1 at the TAD has been implicated in its role in autoimmune and antipathogen responses (13), although further research and functional analyses are required to determine its mechanism in antimycobacterial defense.

The clinical phenotype in our mutation was most compatible with AD STAT1 deficiency, and functional studies demonstrated STAT1 loss-of-function. Identification of the same mutation in both siblings may indicate germline mosaicism, as both siblings are affected, in the parental gametes, although further investigation would be required to confirm this. Gonadal mosaicism is of higher possibility than gonosomal mosaicism, as both parents are asymptomatic, and they do not have the same mutation identified in their peripheral blood cells. Gene mosaicism has been recognized but reported rarely in patients with inborn errors of immunity (IEI), including X-linked severe combined immunodeficiency, X-linked agammaglobulinemia, hyper-IgE syndrome, X-linked chronic granulomatous disease, autoimmune lymphoproliferative disease, Blau syndrome, and cryopyrin-associated periodic syndrome, most of which have been somatic mosaicisms (14–19). In particular, germline mosaicism has been described in a case report previously, which was associated with a gain-of-function mutation of STAT1 (20). However, a recent study investigating gene mosaicism in families with IEIs reported an incidence of up to 25% overall, including somatic, gonadal, and gonosomal mosaicism, of which 3.3% was gonadal and revertant mosaicism (21), suggesting that gene mosaicism plays an underrecognized but hugely relevant role in patients with IEIs, and such findings warrant further research into the genetic mechanisms underlying the pathogenesis of IEIs and the clinical importance of genetic counseling for families affected.

To date, 22 patients from 14 kindreds with 14 heterozygous mutations have been reported to be associated with AD STAT1 deficiency, the majority of which were shown to be LOF mutations with dominant negative effects. Incomplete penetrance is a typical feature and has been quoted to be 88%, and good outcomes with mortality of 0% (12). However, asymptomatic carriers may also display functional defects (22). Although this report is limited by the small number of patients restricted to a single family and the lack of *in vivo* functional and animal model experiments, the novel finding of loss-of-function mutation in the TAD of *STAT1* confirmed by the GAS reporter assay for both siblings further contributes to the current knowledge on STAT1 deficiency. In a recent systematic review, the clinical spectrum of AD STAT1 deficiency is diverse and most commonly manifests as MSMD, of which *Mycobacterium bovis* from BCG was the most common pathogen (23), and multifocal osteomyelitis is also a relatively common reported feature (24–26). However, increased susceptibility to viral infections has also been reported in rare instances (24). This is in contrast to AR complete and partial STAT1 deficiency, in which recurrent viral infections is a common feature in addition to susceptibility to intracellular bacteria (12, 23). This reflects impaired IFN-γ signaling with preserved IFN-α and IFN-β signaling in AD STAT1 deficiency. The most common anatomical site in AD STAT1 deficiency involved was the bone, followed by cutaneous and disseminated disease. When considering disease involvement of both AD STAT1 deficiency and AR STAT1 deficiency together, however, a recent study found that among 64 patients with AR or AD STAT1 deficiency, the most common sites were disseminated disease for 44.4%, followed by cutaneous disease for 41.3% and then bone disease for 39.7% (24). This is not surprising, as AR partial or complete STAT1 deficiency often has more severe clinical presentation compared to AD STAT1 deficiency. Treatment recommendations differ significantly within the spectrum of STAT1 deficiency due to the difference in clinical severity, in which hematopoietic stem cell transplant (HSCT) is recommended for those with AR complete STAT1 deficiency, while antimicrobial treatment is generally adequate for AR partial and AD STAT1 deficiency (4).

Aside from MSMD, BCGiosis can be caused by a myriad of other immunodeficiencies, and therefore, its clinical manifestation should raise concern for underlying immunodeficiency, followed by relevant investigations that should be arranged. In a recent single center review in Singapore, patients with this presentation was ultimately diagnosed with a multitude of underlying IEIs, including SCID, combined immunodeficiency (CID), MSMD, and anhidrotic ectodermal dysplasia with primary immunodeficiency (EDA-ID) (27). In terms of workup for immunodeficiency, the initial abnormal DHR result in our patient corroborated a previous study’s finding that acute infection can affect the reliability of DHR (28).

For this family, the timely diagnosis and implementation of family screening, with the help of WES, was crucial in the identification of the same mutation in his younger sister, for whom exposure to BCG was successfully prevented by omission of the mandatory BCG vaccine that is given as part of the Childhood Vaccination Program in Hong Kong. Under this vaccination program, infants with undiagnosed inborn errors of immunity can be exposed to BCG that is routinely given at birth. The year 2021 marked the commencement of IEI screening, with the implementation of newborn screening for severe combined immunodeficiency (NBS-SCID) by TREC and KREC analysis, in Hong Kong. With the growing trend of newborn screening programs for inborn errors of immunity globally, the importance and potential of screening programs for diagnoses of a broader range of IEIs is expected to be recognized and considered alongside other ethical, socioeconomical, and logistical implications.

In conclusion, the clinical presentation of BCGiosis should raise concern for an underlying immunodeficiency. STAT1 LOF disease should be considered in young children presenting with MSMD. Early genetic diagnosis can provide families with appropriate genetic counseling and medical interventions. Due to the possibility of parental germline mosaicism, genetic testing for siblings should be considered even when parents tested negative. Phenotypic classification and functional analyses of STAT1 LOF mutations play a pivotal role in guiding treatment decisions.

## Data Availability

The datasets presented in this study can be found in online repositories. The names of the repository/repositories and accession number(s) can be found in the article/**Supplementary Material**.
