# Supplementary material for: Case report: A novel de novo germline loss-of-function mutation in the STAT1 transactivation domain in two Chinese siblings, with the elder sibling presenting with multifocal Bacillus Calmette–Guerin osteomyelitis
Source: Front Immunol. 2025 Jan 7;15:1504816. doi: 10.3389/fimmu.2024.1504816 (PMC11747594; doi:10.3389/fimmu.2024.1504816)
Supplement: Supplementary file 1 [file DataSheet1.docx]

Supplementary Material

**METHODS AND MATERIALS**

# Ethics

This study was performed in line with the principles of the Declaration of Helsinki. Approval was granted by the University of Hong Kong Institutional Review Board (UW 08-301). Written informed consent was obtained from the parents of the patients. The authors affirm that human research participants provided informed consent for publication of the images in Figures 1a, 1b, 1c, 1d, 1e, 4a, 4b, 4c, 4d, 4e, and 4f.

# Methodology for whole exome sequencing and Sanger sequencing

Whole exome sequencing (WES) was performed for patient 1 by isolation of genomic DNA from peripheral blood. The identified mutation was confirmed subsequently by targeted gene Sanger sequencing using polymerase chain reaction (PCR). The mutations, identified by bioinformatics analysis, were described with reference to the Human Genome Variation Society (HGVS) nomenclature.

# Methodology for cytokine release test

The cytokine release test was performed by culturing peripheral blood mononuclear cells (PBMC) with IL-12 or IFN-γ, with or without phytohemagglutinin (PHA) or lipopolysaccharide (LPS), followed by measurement of cytokine release.

# Methodology for gamma activated sequence (GAS) luciferase reporter assay

A luciferase assay was conducted to evaluate the STAT1 S701F mutation, following the previously report (Ref. DOI: doi: 10.3324/haematol.2013.083741). In brief, STAT1 deficient U3C cells were seeded in 96-well plates (1 × 104 cells/well) and transfected with reporter plasmids (Cignal Interferon gamma-activated sequence (GAS) Reporter Assay Kit, QIAGEN, Hilden, Germany) and plasmid DNA carrying either the wild type (WT) or each STAT1 variant, using Lipofectamine LTX (Thermo Fisher Scientific, Waltham, MA) in accordance with the manufacture’s protocol. At 24 hours post transfection, the cells were treated with 10 or 1000 IU/mL of IFN-γ (R&D systems, Minneapolis, MN) for 16 hours and subjected to a luciferase reporter assay. Luciferase assays were performed using the Dual-GloR Luciferase Assay System (Promega). The GAS transcriptional activity was determined by normalizing the values against the Firefly/Renilla luciferase luminescence ratio. Three individual experiments were performed in triplicate.
